# Knowledge and indulgence in substsance abuse among adolescents in Anambra state, South-East Nigeria

**DOI:** 10.4314/ahs.v22i1.29

**Published:** 2022-03

**Authors:** Ogochukwu Ofiaeli Chioma, Ifeoma Udigwe Bridget, Chizalu Ndukwu Ifeyinwa, Obiageli Emelumadu Fidelia

**Affiliations:** 1 Department of Paediatrics, Faculty of Medicine, Nnamdi Azikiwe University, Anambra state, Nigeria; 2 Department of Community Medicine, Faculty of Medicine, Nnamdi Azikiwe University, Anambra state, Nigeria

**Keywords:** Drug abuse, adolescence, knowledge, Nigeria

## Abstract

**Background:**

Substance abuse is a growing societal problem with adolescents being at increased risk. The few studies in Nigerian adolescents have not factored in their knowledge base with regard to the concept of substance abuse.

**Objectives:**

This study determined the indulgence in and knowledge of substance abuse and associated factors among adolescents.

**Methods:**

This was a questionnaire based study involving 10 to 19year olds recruited from an adolescent summer camp in Anambra state, South-East Nigeria.

**Results:**

The data of 276adolescents was analyzed, male–91, 33%, M: F = 1: 2. Mean age was 16.4 ± 1.4years. 13.8% (38) accepted they had abused substances in the past; 74.3% (205) had the correct knowledge of the meaning of substance abuse; 10.1% (28) admitted taking substances for pleasure. The substances taken included Alcohol (67.9%), Cigarette (25.0%), Tramadol (10.7%), Cocaine (7.1%), among others. Multiple substances were taken 28.6% of the time.

Age category had no significant association with the abuse of substances (X2–2.656, p = 0.282). Stratified by age category, gender had a significant association with substance abuse in Late adolescence (n = 11; M–9, 81.8%; F–2, 18.2%; X2 = 6.893, p = 0.016) but not Mid-adolescence (n = 27; M – 10, 37.0%; F – 17, 63.0%; X2 = 0.749, p = 0.500).

**Conclusion:**

An unacceptable proportion of the adolescents were already exposed to substances/drugs in spite of having suboptimal knowledge. Adolescents need to be educated on substance abuse and its dangers in order to curb this in the society.

## Introduction

Adolescence is one of the most rapid phases of human development and experiences in this phase of life impact heavily on health and later life.^1^ Traditionally, adolescent age has been defined as 10 – 19 years, youth 15 – 24 years and young people cover the 10 – 24 years age range.^2^ According to a 2016 report, there are about 1.2 billion adolescents aged 10 – 19 years globally making up 16% of the world's population.^3^ 1n sub-Saharan Africa, adolescents make up the greatest proportion of the region's population comprising 23%.^3^ In Nigeria, over 30 million are aged 10 – 19 years and about a third of the total population of Nigerians are 10 – 24 years old.^4^

Substance abuse refers to the detrimental use of psychoactive substances, including alcohol and illicit drugs, which can lead to a number of dependence syndromes/disorders.^5^ In 2016, about 275 million people globally age 15–64 years used drugs at least once.^6^ In 2017, drug use estimates were higher than previously anticipated due to additional drug survey information from Nigeria and India; it was estimated in the same year that 42 million years of healthy life had been lost globally due to drug use.^7^

An adolescent's brain is still vulnerable and immature and thus with reduced capacity to assess situations and make sound decisions.^8^ As a result, most people are likely to begin abusing drugs including alcohol, tobacco, prescription medications and illegal substances in adolescence and young adulthood with development of attendant sequelae.^9^ Globally, greater than a quarter (26.5%) of all 15–19year olds are current drinkers amounting to 155 million adolescents, most of whom are males.^10^ A national drug survey in Nigeria in 2017 found that 14.3 million people aged 15–64 years used drugs; one in four of such drug users was a female.^11^ Various kinds of substances or drugs are abused globally and in adolescence.^9^,^11^ These include but are not limited to alcohol, tobacco, cannabis, opioids, cocaine among others.^12^ The hazardous use of alcohol and drugs are leading risk factors for population health worldwide and have direct impact on many health-related targets of Sustainable Development Goals (SDGs), including those for maternal and child health, non-communicable and infectious diseases like HIV, viral hepatitis, tuberculosis, mental health, injuries and poisonings.10 Annually, 3.3 million deaths worldwide are due to harmful consumption of alcohol.5 Out of the 11 million injection drug users globally, 1.3 million live with HV, 5.5 million have hepatitis C and 1.3 million have both.^5^ In the US, 4.8% of deaths in children and adolescents in 2016 were due to drug over dose and poisoning.^13^

In adolescence, alcohol is the most commonly abused substance.^9^ This is followed by cannabis which is the most commonly used drug and then by the non-medical use of prescription opioids.^9^,^11^ These findings are similar to findings in adult age group.^14^ The drug survey carried out in Nigeria in 2017 placed her among the countries with the highest prevalence of the use of cannabis and non-medical opioids (tramadol) globally.^7^ Early initiation to substance use during adolescence contributes to increasing rates of substance abuse disorders. In Nigeria, average age of initiation to cannabis and opioid is 19 years and 21 years respectively.^11^

Various prevalence rates have been given for substance abuse among adolescents in Nigeria. Idowu et al^15^ found that 26.3% of school children from selected secondary schools in an urban community in Oyo state, South – West Nigeria were abusing drugs; tramadol was the most abused substance. In Abakaliki South - East Nigeria, Anyanwu et al^16^ reported that 32.9% adolescents, secondary school students, abused substances with alcohol being the most common substance abused. In Northern Nigeria, Idoko et al^17^ reported that 21% of secondary school adolescents in Kaduna state engaged in substance abuse with the earliest age group of initiation being 5–9 years. Despite these, there is still a dearth of information on substance abuse in adolescents in Nigeria. None of these studies factored in the knowledge base of these adolescents with regard to the concept of substance abuse.

To prevent drug abuse, it is recommended that protective factors such as parental support need to be enhanced while risk factors such as deviant behaviours be reversed.^18^–^20^ So far, Nigeria's response to the problem of substance abuse in adolescents has been reported to be suboptimal as the National Drug Law Enforcement Agency (NDLEA) and National Agency for Food and Drug Administration and Control (NAFDAC) are not fully supported to function in this role.^20^ An evidence based approach has been suggested as an effective way to tackle the problem of substance use and abuse in adolescents in Nigeria.^20^ By this, risks and protective factors associated with substance abuse are identified through research and preventive strategies structured according to these factors.^21^

This study was thus carried out to determine the indulgence of adolescents in the abuse of substances/drugs as well as their knowledge of substance abuse. Socio-demographic factors associated with substance use such as age and gender were analyzed. Other factors that may be associated with substance use such as recreational use of substances was analyzed. The participants' response to the problem of substance abuse was also sought for.

## Methods

### Ethical considerations

Confidentiality of the participants was ensured at all times. Participation was entirely voluntary and free of charge. The participants were free to withdraw from the research at any point in the study without repercussion. Ethical approval for the study was granted by the Nnamdi Azikiwe University Teaching Hospital (NAUTH) Ethical Review Committee. Consent was also obtained from the organizers of the youth camp, who served as guardians for the participants for the duration of the camp. Consent/assent as applicable by age was obtained from each adolescent before recruitment into the study. No harm came to any child for participating in this study.

### Data collection and analysis

This was a descriptive cross-sectional study involving 276 adolescents aged 10 to 19 years recruited from an adolescent summer/long vacation camp in August of 2019 at Ozubulu, Ekwusigo Local Government Area (LGA) of Anambra state, South-East Nigeria. Adolescents attending this camp cut across junior and seniorlasses of secondary schools for both males and females in 4 neighbouring LGAs in Anambra state, South-East Nigeria - Ekwusigo LGA, Nnewi North LGA, Nnewi South LGA and Ihiala LGA.

A semi structured pretested self-administered questionnaire was used to collect data on involvement in and knowledge of substance abuse. Data was collected consecutively. The outcome variable was ‘abuse of substances’. This was determined from the answers provided to questions in the questionnaire. Explanatory variables included socio-demographic variables such as age and gender and details on recreational use of substances. The nature of response of the recruited adolescents to a perceived problem of substance abuse was also analyzed. Chi square was the test of association. Data was analyzed using SPSS 21 and the level of significance was set at p ≤ 0.05.

A sensitization on substance abuse and its sequelae was carried out after data collection. Misconceptions and myths concerning substance use and misuse, held by the adolescents, were addressed at the end of the exercise. All costs were borne by the researchers.

## Results

The data of 276 adolescents was analyzed, M: F = 1: 2. Mean age was 16.4 ± 1.4 years, median age = 16 years. Early adolescence (10–13years): n = 5; Mid-adolescence (14–17years): n = 214; Late adolescence (18–19years): n = 57. (Table 1)

**Table 1:** Age and gender distribution of the study subjects

Age category	Gender		Total (%)

	Male (%)	Female (%)	
**Early adolescence** **10 – 13 years**	0 (0.0)	5 (100.0)	5 (100.0)
**Mid adolescence** **14 – 17 years**	64 (29.9)	150 (70.1)	214 (100.0)
**Late adolescence** **18 – 21 years**	27 (47.4)	30 (52.6)	57 (100.0)
**Total**	91 (32.7%)	185 (67.3)	276 (100.0)

10.1% (28) of the study participants admitted taking substances for pleasure; 2.5% (7) acknowledged injecting themselves with drugs (Table 2). The substances taken included Alcohol (67.9%), Cigarette (25.0%), Tramadol (10.7%), Cocaine (7.1%), among others. Multiple substances were taken 28.6% of the time.

**Table 2:** Substance use behavior

Variable	Frequency		Total (%)

	Yes (%)	No (%)	
**Have you ever** **injected yourself** **with drugs?**	7 (2.5)	269 (97.5)	276 (100.0)
**Has your friend** **ever injected you** **with drugs?**	4 (1.4)	272 (98.4)	276 (100.0)
**Have you ever** **taken substances** **for pleasure?**	28 (10.1)	248 (89.9)	276 (100.0)
**Have you ever** **abused** **drugs/substances?**	38 (13.8)	238 (86.2)	276 (100.0)

**Figure 1:**
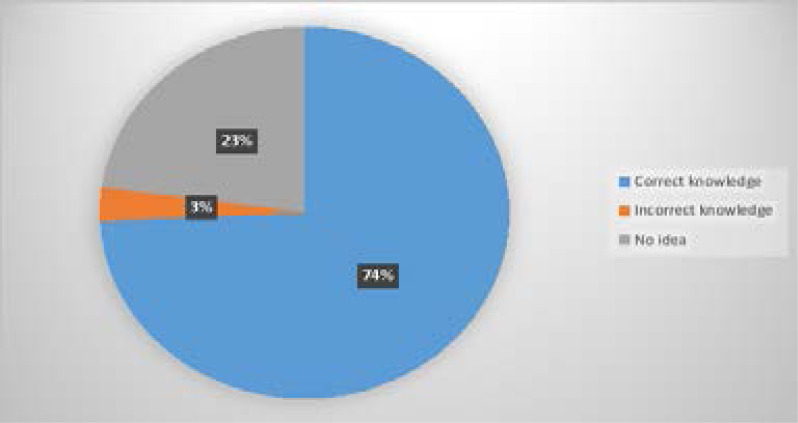
Knowledge of Substance Abuse

74.3% (205) of the respondents had the correct knowledge of what substance abuse was (Figure 1). 13.8% (38) of the respondents accepted they had abused substances in the past and 3.6% (10) believed they had a substance abuse problem. Age category had no significant association with the abuse of substances (X2 – 2.656, p = 0.282, 99% CI – 0.270 to 0.293). Gender had a significant association with abuse of substances (M – 19, 50.0%; F – 19, 50.0%; X2 = 5.782, p = 0.016).

Stratified by age category, gender had a significant association with substance abuse in Late adolescence (n = 11; M – 9, 81.8%; F – 2, 18.2%; X2 = 6.893, p = 0.016) but not Mid-adolescence (n = 27; M – 10, 37.0%; F - 17, 63.0%; X2 = 0.749, p = 0.500) – Table 3.

**Table 3:** Gender association with substance abuse stratified by age category

Age category	Gender	Have you ever abused drugs/substances		Total (%)	X^2^	P value

		Yes (%)	No (%)			
**Early adol.** **10–13** **years**	Male	0 (0.0)	0 (0.0)	0 (0.0)		
	Female	0 (0.0)	5 (100.0)	5 (100.0)	-	-
**Mid adol.** **14–17** **years**	Male	10 (15.6)	54 (84.4)	64 (100.0)		
	Female	17 (11.3)	133 (88.7)	150 (100.00	0.749	0.500
**Late adol.** **18–21** **years**	Male	9 (34.6)	17 (65.4)	26 (100.0)		
	Female	2 (6.7)	28 (93.3)	30 (100.0)	6.893	* **0.016**
**Total**	Male	19 (21.1)	71 (78.9)	90 (100.0)		
	Female	19 (10.3)	166 (89.7)	185 (100.0)	5.782	* **0.016**

Adol. – Adolescence

Chi square (X^2^) test of association.

* significant

Among those who had abused substances, 26.3% (10) believed they had a substance abuse problem, 76.3% (29) had the correct knowledge of what substance abuse was and 67.9% (19) acknowledged recreational use of substances. Recreational use of substances had a significant association with the abuse of these substances among the respondents (X2 = 76.788, p = <0.001) – Table 4.

**Table 4:** Association between recreational use of substances and abuse of drugs/substances

Recreational use of substances	Abuse of substances		Total (%)	X^2^	P value

	Present (%)	Absent (%)			
**Present**	19 (67.9)	9 (32.1)	28 (100.0)		
**Absent**	19 (7.7)	229 (92.3)	248 (100.0)	76.788	* **<0.001**
**Total**	38 (13.8)	238 (86.2)	276 (100.0)		

* significant, p < 0.05

Amidst the respondents who believed they had a substance abuse problem, 60% (6) sought for help; 40% (4) sought help from a doctor/medical facility, 16.7% (1) went to a prayer house. 83.3% (5) of the respondents who tried to get help for their problem claimed they got relief from the intervention received.

## Discussion

Substance abuse is a great challenge to the health and well-being of adolescents. Our study reveals that 14 out of every 100 adolescents aged 10 – 19 years abused substances. Though this is lower than what was reported by Idowu et al^15^ in Oyo state, Anyanwu et al16 in Ebonyi state and Idoko et al^17^ in Kaduna state, Nigeria, it is still unacceptably high. The lower prevalence found in our study may be accounted for by the studied age group. Adolescents between 10 – 19 years comprised our study population, excluding young adults. In addition, increasing sensitization on the subject matter over the years could also be contributory. Despite this, a lot still needs to be done to bring this prevalence much lower. Our finding also lays credence to the fact that a good number of cases of substance use disorder diagnosed in adulthood could have their onset in childhood/adolescence. This is worrisome as they still have a long life span of possible continued exposure to these substances and the attendant complications.

Multiple substances were abused by respondents in this study with alcohol being the most abused. This is worrisome as a great proportion of deaths worldwide are attributed to alcohol consumption.^5^ Gender had a significant association with substance abuse in late adolescence in this study with more males indulging in the habit. This is comparable to the reports given both globally and from Nigeria of adolescent males indulging more in alcohol consumption compared to their female counterparts.^10^,^11^ This can be explained by the greater level of youthful exuberance and risk taking displayed by adolescent males when compared to adolescent females.

The few studies done on Nigerian adolescents have not factored in their knowledge base with regard to the concept of substance abuse. Three-quarters of the respondents from this study had adequate knowledge. This value really needs to be improved upon. Among respondents who abused substances, three-quarters also had right knowledge. This finding could imply a conscious and willful indulgence in substance abuse and might serve as a poor indicator for continued abuse in adulthood with increased risk for attendant sequelae. Among the adolescents abusing substances, two-thirds admitted using these recreationally. This might explain how this poor habit was formed in the 1^st^ place. Recreational use of substances had a significant association with the abuse of substances in this study.

A quarter of those who abused substances believed they had a substance abuse problem and 60% of them sought for help; most of whom claimed they got relief from the interventions they received. This is encouraging and buttresses the need for improved knowledge in adolescents which will serve as the 1st point of empowerment towards freedom from substance abuse. This can be achieved through school programs and peer groups.^20^,^21^ Families can also be empowered to support adolescents better in avoiding substance use / abuse or getting out of substance abuse if it has already developed. 20,21 These measures could serve as primary points in dealing with substance abuse in adolescents.

## Conclusion

An unacceptable proportion of the adolescents were already exposed to substances/drugs in spite of having suboptimal knowledge. Recreational use of substances had a significant association with the abuse of substances. Adolescents need to be educated on substance abuse and its dangers. This will go a long way to curb this in the society.
